# Spread of Certain Diseases

**Published:** 1874-11

**Authors:** 


### SPREAD OF CERTAIN DISEASES.

It is to be regarded as a settled fact that diseases of a contagious nature are caused and spread by influences largely within the sphere of our control. This fact has recently been strongly urged by Dr. Symes Thompson, a well-known English physician, in a lecture delivered by him in London. Every form of infectious fever, he asserts, has its idiosyncrasy. Thus, enteric fever and cholera tend chiefly to disseminate themselves through water passing into wells and fountains of daily supply; scarlet fever hibernates in a drawer, and, after long months, comes forth with some old and cast-aside garment, to be thrown with it around the throat or head of some new victim, and so start thence upon a fresh career; typhus fever crawls sluggishly from hand to hand and mouth to mouth; typhoid fever generates itself where filth, overcrowding and impure habits of life prevail. So well known are those idiosyncrasies, and the means of control, that the existence or spread of such disease is directly attributable to neglect of the most simple laws of prevention.

